# Increased spending on low-value care during the COVID-19 pandemic in Virginia

**DOI:** 10.1093/haschl/qxae133

**Published:** 2024-10-23

**Authors:** Michelle S Rockwell, Sitaram Vangala, Jillian Rider, Beth Bortz, Kyle Russell, Marcos Dachary, Lauryn Walker, A Mark Fendrick, John N Mafi

**Affiliations:** Department of Family and Community Medicine, Virginia Tech Carilion School of Medicine, Roanoke, VA 24016, United States; The Department of Medicine Statistics Core, David Geffen School of Medicine at UCLA, Los Angeles, CA 90024, United States; Virginia Health Information, Richmond, VA 23219, United States; Virginia Center for Health Innovation, Henrico, VA 23233, United States; Virginia Health Information, Richmond, VA 23219, United States; Milliman MedInsight, Seattle, WA 98101, United States; Virginia Center for Health Innovation, Henrico, VA 23233, United States; Center for Value-Based Insurance Design, University of Michigan School of Medicine and School of Public Health, Ann Arbor, MI 48109, United States; Division of General Internal Medicine and Health Services Research, David Geffen School of Medicine at UCLA, Los Angeles, CA 90024, United States; RAND Health Care, RAND Corporation, Santa Monica, CA 90401, United States

**Keywords:** low-value care, high-value care, health care costs, health equity, socioeconomic disadvantage, area deprivation index

## Abstract

Characterizing the value and equity of care delivered during the COVID-19 pandemic is crucial to uncovering health system vulnerabilities and informing postpandemic recovery. We used insurance claims to evaluate low-value (no clinical benefit, potentially harmful) and clinically indicated utilization of a subset of 11 ambulatory services within a cohort of ∼2 million Virginia adults during the first 2 years of the pandemic (March 1, 2020–December 31, 2021). In 2020, low-value and clinically indicated utilization decreased similarly, while in 2021, low-value and clinically indicated utilization were 7% higher and 4% lower, respectively, than prepandemic rates. Extrapolated to Virginia's population of insured adults, ∼$1.3 billion in spending was associated with low-value utilization of the 11 services during the study period, with 2021 spending rates 6% higher than prepandemic rates. During March 1, 2020–December 31, 2021, low-value and clinically indicated utilization were 15% and 16% lower, respectively, than pre-pandemic rates among patients with the greatest socioeconomic deprivation but similar to prepandemic rates among patients with the least socioeconomic deprivation. These results highlight widening healthcare disparities and underscore the need for policy-level efforts to address the complex drivers of low-value care and equitably redistribute expenditures to services that enhance health.

## Teaser

Characterizing the value and equity of care delivered during the COVID-19 pandemic is crucial to uncovering health system vulnerabilities and informing postpandemic recovery. This study evaluated utilization and spending on low-value care delivered in Virginia during the first two years of the COVID-19 pandemic. Did the Virginia health system adapt to reduce low-value care while prioritizing clinically indicated and high-value care? Did the delivery of low-value care differ in rural vs. urban areas and by socioeconomic status? Understanding these care patterns can inform efforts to improve health and reduce disparities in Virginia.

Low-value care—health care that offers no net clinical benefit in specific scenarios—comprises up to 20% of US health care spending and is a source of preventable harm. Individuals from disadvantaged and historically marginalized communities may be particularly impacted by low-value care and its harmful sequellae.^1,2^ Utilization of low-value care has proven persistent and challenging to de-implement.^2^

Declared by the World Health Organization in March 2020, the COVID-19 pandemic introduced an unprecedented opportunity to disrupt low-value care. We and others were optimistic that the health system would adapt to reduce low-value care while prioritizing clinically indicated and high-value COVID and non-COVID care.^3,4^ Unfortunately, early reports suggest that the delivery of low-value care generally returned to near prepandemic rates within months of the pandemic's onset.^5-7^ These studies primarily focused on the first year of the pandemic and did not evaluate implications on spending or socioeconomic disparities. Characterizing the value and equity of care delivered during the COVID-19 pandemic is crucial to uncovering health system vulnerabilities and informing postpandemic recovery.

In the present study, we used one of the largest state-based all-payer claims databases (APCD) to evaluate utilization and spending for a subset of ambulatory services in Virginia during the first 2 years of the COVID-19 pandemic. We also assessed variation in low-value and clinically indicated utilization based on the socioeconomic status and rurality of the areas in which patients resided. We hypothesized that the delivery of low-value care rebounded to prepandemic levels to a lesser extent than clinically indicated care during 2021 and that both low-value and clinically indicated care were disproportionately reduced among Virginians living in areas with the greatest socioeconomic deprivation and those living in rural areas throughout 2020 and 2021.

## Study data and methods

In this retrospective cohort study, we used the Virginia APCD to analyze a subset of 11 ambulatory health services delivered to adult patients during 2019–2021. Based on the use of de-identified, publicly available data, the Institutional Review Board (IRB) of Carilion Clinic determined that this project did not require IRB oversight.

## Data sources and study population

The Virginia APCD is comprised of medical and pharmaceutical claims for ∼5.5 million patients insured by Medicaid, Medicare Advantage, Traditional Medicare, dual (Medicare–Medicaid), and commercial payers representing 75% of the state's population. We established a study cohort of patients ≥18 years of age and continuously enrolled for ≥36 months prior to 2019, 2020, and 2021, in addition to three sensitivity cohorts (Supplementary Material A). We obtained utilization, spending, and enrollment data aggregated at the population level by demographics (sex, age bracket, socioeconomic status, payer, and rurality). Socioeconomic status was approximated via area deprivation index (ADI), which incorporates the domains of income, education, employment, and housing quality to quantify area-level socioeconomic deprivation on a 1–100 scale (Supplementary Material B).^8^ Rurality was assigned using USDA rural–urban commuting area (RUCA) codes derived from patient zip codes, with RUCA 1–3 = urban and 4–10 = rural.^9^

We evaluated claims for a subset of 11 ambulatory preventive screenings, diagnostic tests, and preoperative services selected by a stakeholder taskforce established for the Virginia Center for Health Innovation (VCHI) Smarter Care Virginia initiative and previously studied by our team and others.^10-12^ The services included electrocardiogram (EKG) screening, cardiac stress testing, vitamin D screening, imaging for low back pain, headache, syncope, or eye disease, imaging for dizziness in the emergency department, and preoperative laboratory, cardiac, or pulmonary testing.

### Utilization and spending analysis

Using the Milliman MedInsight Health Waste Calculator, claims for the 11 services were classified as low-value or clinically indicated as described previously^10,11,13^ and in Supplementary Material C. We determined age and sex-adjusted monthly utilization rates for low-value and clinically indicated service claims per 1000 patients and assessed total annual spending (payer + patient) for low-value utilization. Spending data were adjusted for inflation using the US Bureau of Labor Consumer Price Index-Medical with 2021 as the reference year, extrapolated to the Virginia population of insured adults (6 million), and adjusted for payer mix (Supplementary Material D).

To maximize interpretability in light of stable prepandemic trends, we compared observed vs prepandemic utilization rates using rate ratios (RR): (*the number of service claims in 2020 or 2021/the number of patients in 2020 or 2021*) divided by (*the number of service claims in 2019/the number of patients in 2019*) monthly and annually for low-value and clinically indicated utilization. We stratified RR findings by area-level socioeconomic status (ADI quartile) and rurality (urban, rural). SPSS v27.0 was used for the analysis.

## Study results

Our study cohort included 1 969 866 patients (mean age: 59.5 years, 56% female). Sociodemographic characteristics of the cohort remained relatively stable between 2019 and 2021 (Supplementary Material E). The proportion of patients covered by Medicaid increased and commercial payers decreased during the study period, reflecting state and national trends during the same timeframe.

Of >40 million claims for the subset of ambulatory services, 23% were classified as low-value in 2019 vs 24% in 2020 and 26% in 2021. Low-value utilization rates for each of the 11 services are shown in Supplementary Material F. The greatest low-value utilization rates were observed for preoperative laboratory tests (179 claims/1000 patients), EKG screening (102 claims/1000 patients), and imaging for eye disease (66 claims/1000 patients).

**Figure 1.**
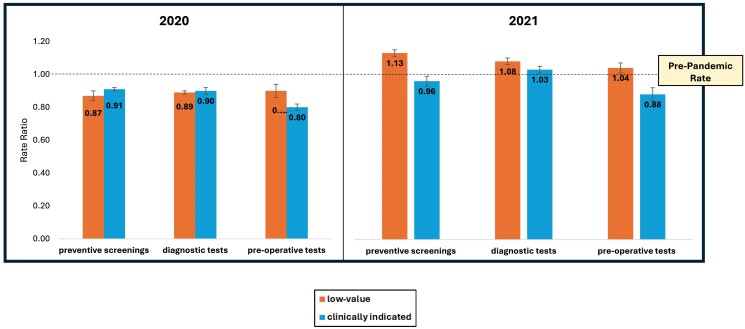
Low-value and clinically indicated utilization of preventive screenings, diagnostic testing, and preoperative testing services in 2020 and 2021 vs prepandemic rates. Abbreviation: APCD, all-payer claims database.

Compared with prepandemic rates, low-value utilization decreased more than clinically indicated utilization at the start of the pandemic (0.45 [0.44–0.47] vs 0.51 [0.50–0.52], *P* = 0.004). However, low-value utilization exceeded clinically indicated utilization vs prepandemic rates in June 2020 and throughout 2021 (Supplementary Material G). Utilization of the selected services decreased similarly to 11% (low-value) and 13% (clinically indicated) beneath prepandemic rates in 2020 (Figure 1). However, in 2021, while clinically indicated utilization remained 4% lower than prepandemic rates, low-value utilization exceeded prepandemic rates by 7% in 2021 (Figure 1). There were no service type differences in low-value utilization, but clinically indicated preoperative services were utilized at significantly lower rates than preventive screenings and diagnostic tests (Figure 1).

In our study cohort, spending for low-value utilization of the studied ambulatory services totaled $618 451 006 in 2020–2021. Extrapolated to the Virginia population of 6 million insured adults and adjusted for payer mix, an estimated $2 billion in spending was associated with low-value utilization of 11 ambulatory services during 2019–2021, with $1.3 billion during the pandemic (January 3, 2020–December 5, 2021). Low-value spending exceeded prepandemic rates by 6% in 2021 (Supplementary Material H).

### Stratified analyses

**Figure 2.**
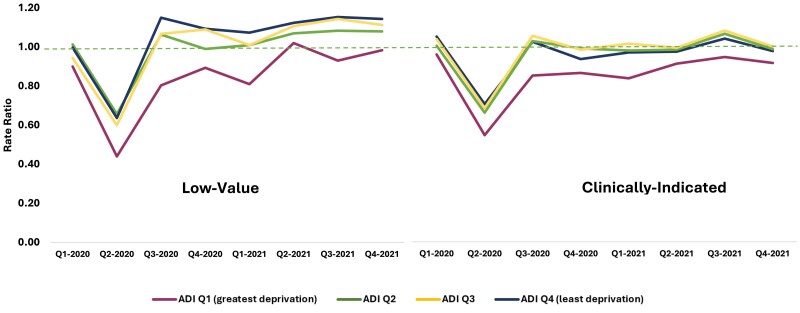
Low-value and clinically indicated utilization of preventive screenings, diagnostic testing, and preoperative testing services in March 1, 2020–December 31, 2021 vs prepandemic rates, stratified by ADI quartile. Quartile 1 (Q1) = greatest deprivation, Quartile 4 (Q4) = least deprivation. The dotted line indicates 100% of prepandemic rates (ie, observed = prepandemic). Source: Authors’ analysis of data from the Virginia APCD using the Milliman MedInsight Health Waste Calculator to categorize low-value and clinically indicated services. Abbreviations: ADI, area deprivation index; APCD, all-payer claims databases.

Low-value utilization of the selected ambulatory services was significantly lower than prepandemic rates among patients residing in areas with the greatest socioeconomic deprivation (top ADI quartile) during the first two years of the COVID-19 pandemic (0.85, [0.84–0.86]) compared with those residing in areas with the least deprivation (lowest ADI quartile) (1.04 [1.03–1.04]) (*P* < 0.001) (Figure 2). Low-value utilization of preventive screenings, diagnostic tests, and preoperative services was 23%, 17%, and 10% lower, respectively, than prepandemic rates in Virginia areas with the greatest socioeconomic deprivation compared with the least deprivation (Supplementary Material I).

Similarly, clinically indicated utilization was significantly lower than prepandemic rates among patients residing in areas with the greatest socioeconomic deprivation (top ADI quartile) (0.84 [0.84–0.85]) compared with those residing in areas with the least deprivation (lowest ADI quartile) (0.95 [0.94–0.96]) (*P* < 0.001) during the same time period (Figure 2). Clinically indicated utilization of preventive screenings, diagnostic tests, and preoperative services was 9%, 2%, and 4% lower, respectively, relative to prepandemic rates in Virginia areas with the greatest socioeconomic deprivation compared with the least deprivation (Supplementary Material I).

Low-value and clinically indicated utilization vs prepandemic rates were similar in rural and urban regions of Virginia throughout the study period (Supplementary Material J).

## Discussion

Low-value care is a persistent source of unnecessary spending and potential harm. This analysis of insurance claims for 2 million Virginians showed similar modest declines in low-value and clinically indicated utilization of a subset of ambulatory health services in 2020% and 7% higher than prepandemic rates of low-value utilization in 2021. These findings build upon early pandemic reports^5-7^ to illustrate enduring patterns of low-value care, even during an unprecedented pandemic and major disruption to the typical delivery of health care. Spending for low-value utilization was $1.3 billion during the first two years of the COVID-19 pandemic, which exceeded prepandemic spending by 6% in 2021. Considering the subset of services studied represents only a fraction of low-value services delivered in ambulatory care, the true statewide financial impact of nonevidence-based, potentially harmful health service utilization was substantial.

While it is encouraging that low-value utilization decreased among communities with the greatest socioeconomic deprivation, the parallel 16% decrease in clinically indicated ambulatory service utilization vs prepandemic rates, which aligns with other published reports,^9,10^ suggests compromised access and disparities in care for some Virginians during the pandemic. For example, the significantly greater utilization of preventive screenings in the lowest vs highest ADI quartile may reflect better access to primary care in low socioeconomic deprivation areas. Our observation that rural and urban areas experienced similar changes in clinically indicated and low-value care may reflect the success of efforts to maintain access to care in rural regions, such as effective implementation of telemedicine.

It is unclear why low-value and clinically indicated utilization of the studied services rebounded similarly in 2020, but low-value utilization rebounded to a greater extent than clinically indicated utilization vs expected rates in 2021. The greatest variation in utilization trends was observed between low-value and clinically indicated preoperative services, which includes laboratory, cardiac, or pulmonary testing performed prior to low-risk surgery. One possibility is that clinicians were more likely to order preoperative testing for otherwise low-risk patients after they had experienced COVID-19 infections. Other contributors may have been decreased quality of care amidst the stress and health system strain of the pandemic, increased patient demand, and clinician shortages and burnout. Financial pressures on health systems may have precipitated increased provision of procedures. Further research is needed to improve understanding of the persistent drivers of low-value care and barriers to high-value care.

Taken together, the results of this study highlight the need for continued efforts to promote the delivery of clinically indicated, high-value care among all Virginians while overcoming the complex and resistant drivers of low-value care. The Smarter Care Virginia initiative is one such example. Smarter Care Virginia is an ongoing multimodal partnership between VCHI and six large health systems that uses education, quality improvement training, and peer comparison performance reports to promote de-implementation of low-value care.^14^ Policy-level interventions, tailored value-based insurance programs, or other disinvestment efforts are additional possibilities to ensure the equitable redistribution of expenditures to medical care that enhances population health.^15^

## Limitations

This study has some limitations. Fluctuations in the population occurred during the study period due to job losses, Medicaid expansion, and other causes. The similar trends in enrollment and utilization observed among our study cohort and the sensitivity cohorts (Supplementary Material A) suggest that the influence of population variation and enrollment policy changes on overall results is minimal. Second, like other APCD studies, our analysis excludes some residents (e.g., uninsured individuals), lacks the specificity of patient-level data, and may omit clinical nuance not accessible via insurance claims. Third, although we used established indices to estimate socioeconomic status and rurality at the area level^8,9^; there are recognized strengths and limitations to these measures, with the main limitation being that these are area-level rather than individual-level factors.^16,17^ Finally, we used a convenience sample that may not generalize to other US states and the subset of services studied may not be illustrative of all low-value ambulatory services utilized during the pandemic.

## Conclusion

In contrast to ours and others’ hypothesis that the utilization of low-value ambulatory care would return to prepandemic rates to a lesser extent than clinically indicated care during the second year of the pandemic (2021), our findings show that low-value utilization and spending for a subset of ambulatory services were 7% and 6% higher, respectively, while clinically indicated utilization was 4% lower than prepandemic rates in Virginia during this time period. Importantly, Virginians with the greatest vs least socioeconomic deprivation received disproportionately reduced rates of both low-value and clinically indicated care in 2021. These results highlight widening healthcare disparities and underscore the need for policy-level efforts to address the complex drivers of low-value care and equitably redistribute expenditures to services that enhance health.

## Supplementary Material

qxae133_Supplementary_Data

## Data Availability

Data from the Virginia APCD are available via https://www.vhi.org/apcd/.
