# Correction to: Increased spending on low-value care during the COVID-19 pandemic in Virginia

**DOI:** 10.1093/haschl/qxae166

**Published:** 2024-12-09

This is a correction to: Michelle S Rockwell, Sitaram Vangala, Jillian Rider, Beth Bortz, Kyle Russell, Marcos Dachary, Lauryn Walker, A Mark Fendrick, John N Mafi, Increased spending on low-value care during the COVID-19 pandemic in Virginia, *Health Affairs Scholar*, Volume 2, Issue 11, November 2024, qxae133, https://doi.org/10.1093/haschl/qxae133

In the originally published version of the manuscript there was an error within Figure 1. The corrected version is:

**Figure:**
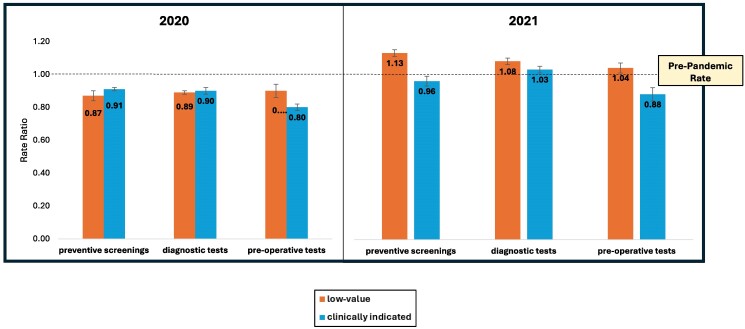


The corrected version has replaced the incorrect:

**Figure:**
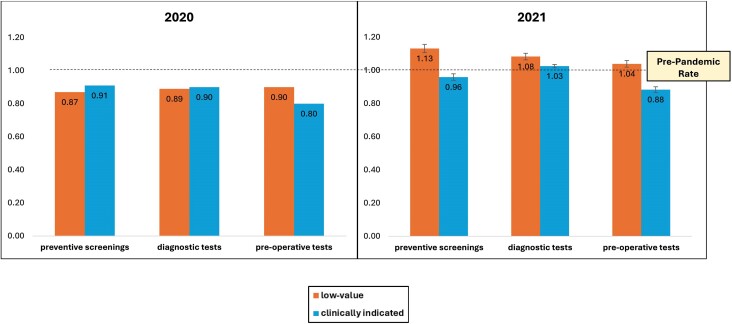


This correction has been made to the paper.

