# Supplementary material for: Increased spending on low-value care during the COVID-19 pandemic in Virginia
Source: Health Aff Sch. 2024 Oct 23;2(11):qxae133. doi: 10.1093/haschl/qxae133 (PMC11549685; doi:10.1093/haschl/qxae133)
Supplement: qxae133_Supplementary_Data [file qxae133_supplementary_data.zip › CLEAN APPENDIX_Low-Value Virginia HA Resub 10.16.24.docx]

**APPENDIX**

**Increased Spending on Low-Value Care During**

**the COVID-19 Pandemic in Virginia**

**A. Study Cohorts: Consort Diagrams, Primary and Sensitivity Cohort Enrollment, and Sensitivity Cohort Utilization Trends**

**B. Procedures for Determining Area Deprivation Index Quartiles**

**C. Specifications for Ambulatory Care Measures**

**D. Procedures for Calculating Statewide Spending**

**E. Sociodemographic Characteristics of the Primary Cohort**

**F. Unadjusted Low-Value Utilization Rates for Preventive Screenings, Diagnostic Testing, and Pre-Operative Testing Services**

**During March 1, 2020 – December 31, 2021**

**G. Low-Value and Clinically-Indicated Screening, Diagnostic, and Pre-Operative Services vs. Pre-Pandemic Rates in Virginia**

**During March 1, 2020 – December 31, 2021**

**H. Spending for 11 Low-Value Screening, Diagnostic, and Pre-Operative Services Delivered in Virginia in 2019 to 2021**

**I. Low-Value and Clinically-Indicated Utilization of Preventive Screenings, Diagnostic Tests, and Pre-Operative Services**

**Among Patients Residing in Areas with the Greatest vs Least Socioeconomic Deprivation in Virginia in 2020-2021**

**J. Low-Value and Clinically-Indicated Utilization of 11 Ambulatory Services vs. Pre-Pandemic Rates in Virginia During 2020**

**and 2021, Stratified by Rurality**

**APPENDIX-A. STUDY COHORTS**

**CONSORT DIAGRAMS**

We established a primary study cohort (continuously-enrolled for >36 months). We also established three pre-specified comparison patient cohorts for sensitivity analyses: 1) continuously enrolled for >24 months; 2) continuously enrolled for >12 months; and 3) not continuously enrolled during the study period.

**
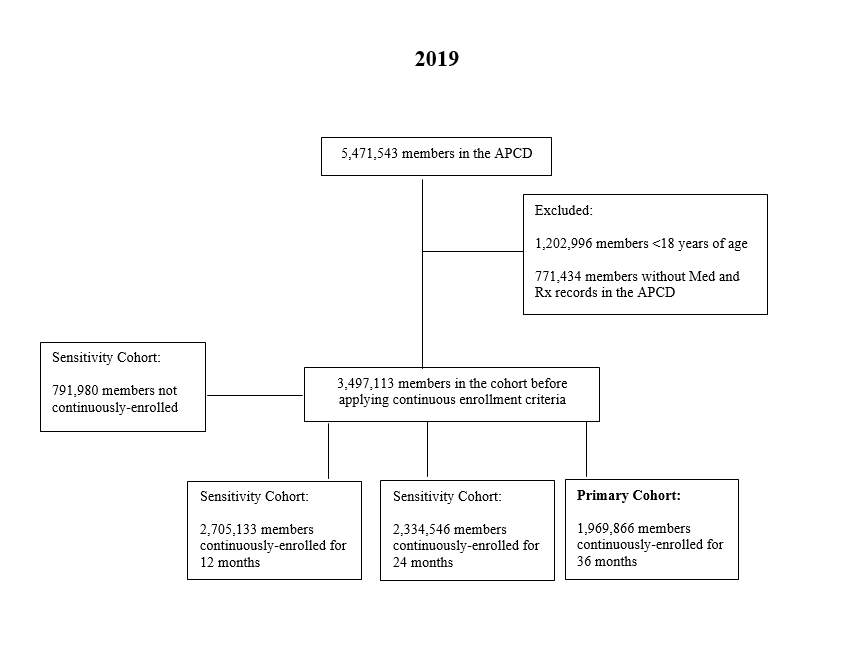
**

**
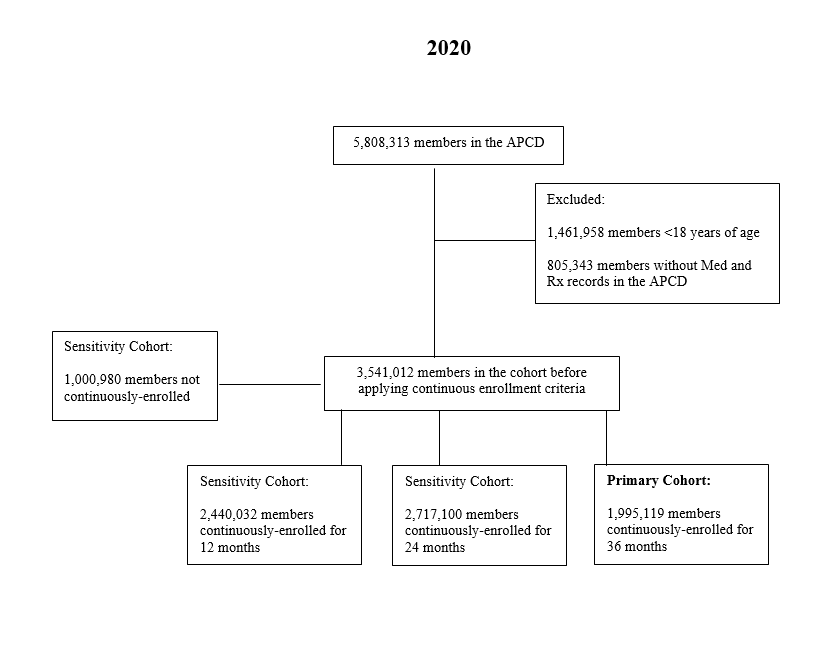
**

**
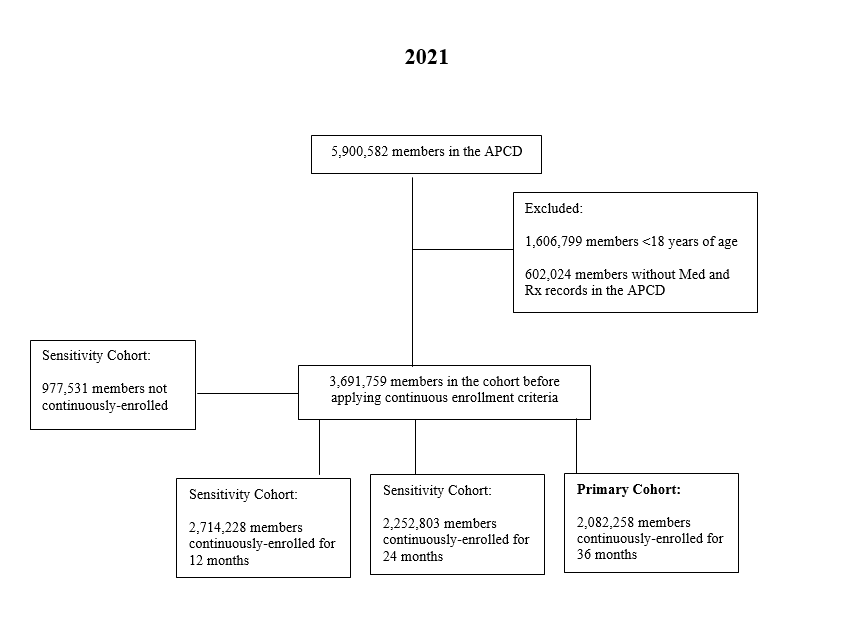
**

**PATIENT ENROLLMENT IN THE 36-MONTH (PRIMARY), 24-MONTH, 12-MONTH, AND**

**NON-CONTINUOUSLY ENROLLED COHORTS**

**SENSITIVITY ANALYSIS: RATE OF LOW-VALUE AND CLINICALLY-INDICATED UTILIZATION DURING 2019-2021**

**SENSITIVITY ANALYSIS: OBSERVED VS. EXPECTED LOW-VALUE AND CLINICALLY-INDICATED UTILIZATION DURING 2020 AND 2021**

**APPENDIX-B.**

**Procedures for Determining Area Deprivation Index Quartiles**

We used the Area Deprivation Index (ADI), developed by Kind et al.^1^ at the University of Wisconsin School of Medicine and Public Health to estimate the socioeconomic conditions throughout Virginia. ADI was selected due to its incorporation of multiple factors that are associated with socioeconomic status but are not available in insurance claims data (e.g. education, employment, and housing) and its association with health outcomes and health care utilization.

We used zip codes for all patients in the cohort to assign ADI scores (40 to 160 points). ADI scores were ranked by percentile (1st-100th percentile). We stratified by quartiles, with patients whose ADI scores were in the top quartile (75th-100th percentile) representing the greatest socioeconomic deprivation and those in the lowest quartile (1st-25th percentile) representing the least socioeconomic deprivation. We then linked patient claims data from the APCD to their respective percentile. The proportion of patients in each quartile was 18% (Q1), 29% (Q2), 26% (Q3), 25% (Q4), and 2% (unknown). Group sizes were similar but not exact since the volume of patients whose ADI scores fell within each quartile varied.

**APPENDIX-C.**

**SPECIFICATIONS FOR AMBULATORY CARE MEASURES**

| **Service Category** | **Measure** | **Criteria for Low-Value Specification** | **Reference(s)** |
| --- | --- | --- | --- |
| Preventive Screenings | ANNUAL EKG SCREENING | EKG screening in low-risk patients without cardiac risk factors. | <https://www.uspreventiveservicestaskforce.org/uspstf/recommendation/cardiovascular-disease-risk-screening-with-electrocardiography> |
|  | VITAMIN D SCREENING | 25-hydroxyvitamin D testing in patients without serious risk factors. | <https://www.choosingwisely.org/patient-resources/vitamin-d-tests/> |
|  | CARDIAC STRESS TESTING | Cardiac screening in asymptomatic patients without significant history or cardiac risk factors. | <https://www.acpjournals.org/doi/10.7326/M14-1225> |
| Diagnostic Tests | IMAGING FOR UNCOMPLICATED EYE DISEASE | Visual-field testing, optical coherence tomography (OCT) testing, retinal imaging of patients with diabetes, neuroimaging or fundus photography, or other routine eye imaging in patients with uncomplicated eye disease. | <https://www.choosingwisely.org/clinician-lists/american-academy-ophthalmology-routine-imaging-for-patients-without-symptoms-or-signs-of-eye-disease/> |
|  | IMAGING FOR ACUTE LOW BACK PAIN | X-ray, CT, or MRI during the first 6 weeks of acute low back pain unless red flags are present. | <https://acsearch.acr.org/docs/69483/narrative/> |
|  | IMAGING FOR UNCOMPLICATED HEADACHE | Electroencephalography within 30 days of headache diagnosis without recent inpatient stay or history of epilepsy, seizure, or other complication. | <https://www.choosingwisely.org/wp-content/uploads/2015/05/ICER_Headache.pdf> |
|  | CT FOR DIZZINESS IN THE ED | CT scan within one day of onset of dizziness in patients without diagnosis of head injury or other head and neck diagnosis or recent inpatient stay. | <https://www.ncbi.nlm.nih.gov/pmc/articles/PMC2676794/> |
|  | IMAGING FOR SYNCOPE | Simple imaging of the carotid arteries for patients without neurological deficit or recent inpatient stay. | <https://www.ncbi.nlm.nih.gov/pmc/articles/PMC3295536/> |
| Pre-Operative Tests | PRE-OPERATIVE LABORATORY STUDIES | Laboratory testing in low-risk patients prior to non-cardiac surgery. | <https://pubs.asahq.org/anesthesiology/article/116/3/522/13033/Practice-Advisory-for-Preanesthesia-EvaluationAn> |
|  | PRE-OPERATIVE EKG, CHEST X-RAY, PFT | EKG, chest x-ray, or pulmonary function test in patients without cardiovascular or pulmonary disease or substantial risk factors prior to low-risk non-cardiac surgery. | <https://pubs.asahq.org/anesthesiology/article/116/3/522/13033/Practice-Advisory-for-Preanesthesia-EvaluationAn> |
|  | PRE-OPERATIVE CARDIAC ECHO OR STRESS TESTING | Echocardiogram or stress testing in patients without cardiovascular or substantial risk factors prior to low-risk non-cardiac surgery. | <https://pubs.asahq.org/anesthesiology/article/116/3/522/13033/Practice-Advisory-for-Preanesthesia-EvaluationAn> |

Further details are shown in Mafi et al.^2^ and Ganguli et al.^3^ . The analysis was limited to claims for which there was sufficient look back history available. In alignment with Healthcare Effectiveness Data and Information Set (HEDIS) measures, up to two months of non-consecutive enrollment gaps per year were permitted. A priori, we excluded data for APCD payer contributors with more than one month of missing data per year. Patients with missing demographic data (<2% of total patients) were included in the analysis.

**APPENDIX-D.**

**PROCEUDRES FOR CALCULATING STATEWIDE SPENDING**

1. We extracted TOTAL PAID data (patient + payer) for 2018, 2019, 2020, 2021 from APCD files (36-m cohort). This represents total actual payments from payers and patients for the ~2 million patient cohort.
2. We adjusted for inflation using US Bureau of Labor Statistics data, with 2021 as the reference year. 2020= 1.2%, 2021= 4.7%.
3. We extrapolated cohort spending to state-level spending using US Census data for Virginia population % insured:

|  | **2019** | **2020** | **2021** |
| --- | --- | --- | --- |
| Virginia adults | 6.6 million | 6.7 million | 6.7 million |
| Uninsured rate | 11.3% uninsured | 8.8% uninsured | 8.2% uninsured |
| **Virginia insured adults** | **5.9 million** | **6.1 million** | **6.2 million** |

1. We adjusted to state-level payer composition.

| ***OUR COHORT*** | **2019** | **2020** | **2021** |
| --- | --- | --- | --- |
| Medicare FFS | 22% | 24% | 24% |
| Medicare Advantage | 25% | 24% | 23% |
| Medicaid | 5% | 7% | 10% |
| Dual | 2% | 2% | 3% |
| Commercial | 44% | 43% | 39% |
| Other | 0% | 0% | 0% |
|  |  |  |  |
| ***VIRGINIA*** | **2019** | **2020** | **2021** |
| Medicare FFS | 15% | 13% | 12% |
| Medicare Advantage | 8% | 10% | 11% |
| Medicaid | 16% | 18% | 21% |
| Dual | 2% | 3% | 3% |
| Commercial | 54% | 50% | 48% |
| Other | 5% | 6% | 5% |

**APPENDIX-E.**

**Sociodemographic Characteristics of Primary Cohort**

|  | **2019**  (n= 1,969,866) | **2020**  (n= 1,995,119) | **2021**  (n=2,082,258) |
| --- | --- | --- | --- |
| **Age (years)** | 59.5 ± 23.9 | 59.4 ± 24.0 | 59.2± 23.5 |
| **Age bands**  **n (%)** 18 to 39  40 to 64  65 to 79  80+  Unknown | 256,082 (13)  650,056 (33)  768,248 (39)  256,082 (13)  39,398 (2) | 279,317 (14)  638,438 (32)  817,997 (41)  259,365 (13)  2 (0) | 291,516 (14)  666,323 (32)  853,726 (41)  270,694 (13)  0 (0) |
| **Sex**  **n (%)** Female  Male  Unknown | 1,099,185 (56)  831,283 (42)  39,397 (2) | 1,133,228 (57)  821,989 (43)  39,902 (0) | 1,195,006 (57)  887,042 (43)  210 (0) |
| **Payer Type**  **n (%)** Commercial  Medicaid  Medicare Advantage  Traditional Medicare  Dual  Unknown | 866,741 (44)  98,493 (5)  433,371 (23)  492,467 (25)  39,397 (2)  19,697 (1) | 857,901 (43)  139,658 (7)  478,829 (24)  478,896 (24)  39,835 (2)  0 (0) | 812,080 (39)  208,188 (10)  499,742 (24)  499,808 (24)  62,440 (3)  0 (0) |
| **ADI^†^**  **n (%)** Quartile 1  Quartile 2  Quartile 3  Quartile 4  Unknown | 354,576 (18)  571,261 (29)  512,165 (26)  492,467 (25)  39,397 (2) | 339,170 (17)  578,585 (29)  538,682 (27)  498,780 (25)  39,902 (2) | 374,806 (18)  603,855 (29)  562,210 (27)  540,087 (26)  1,300 (0) |
| **Rurality**  **n (%)** Urban  Rural  Unknown | 1,674,386 (85)  256,082 (13)  39,397 (2) | 1,695851 (85)  259,366 (13)  14,649 (2) | 1,810,264 (86)  270,694 (14)  1,300 (0) |

^†^= Area Deprivation Index (ADI). ADI Quartile 1= greatest socioeconomic disadvantage

**APPENDIX F. Unadjusted Low-Value Utilization Rates for Preventive Screenings, Diagnostic Testing, and Pre-Operative Testing Services During March 1, 2020 – December 31, 2021**

**APPENDIX G. Low-Value and Clinically-indicated Screening, Diagnostic, and Pre-Operative Services vs. Pre-Pandemic Rates in Virginia During March 1, 2020 – December 31, 2021**

COVID-19 Pandemic Declared by WHO

The dotted line indicates Rate Ratio=1.00, meaning that observed rates = pre-pandemic rates.

**APPENDIX H. SPENDING FOR 11 LOW-VALUE SCREENING, DIAGNOSTIC, AND PRE-OPERATIVE SERVICES DELIVERED IN VIRGINIA IN 2019 TO 2021.**

|  | **Preventive**  **Screenings** | **Diagnostic**  **Tests** | **Pre-Operative**  **Services** | **Total** |
| --- | --- | --- | --- | --- |
| **2019** | $ 90,762,420 | $ 209,111,309 | $ 351,382,048 | $ 651,255,777 |
| **2020** | $ 78,214,738 | $ 208,799,214 | $ 330,235,935 | $ 617,249,887 |
| **2021** | $ 87,248,975 | $ 215,936,303 | $ 386,028,081 | $ 689,213,359 |
|  |  |  |  | **$ 1,957,719,023** |

**APPENDIX I. Low-Value and Clinically-Indicated Utilization of Preventive Screenings, Diagnostic Tests, and Pre-Operative Services STRATIFIED BY Socioeconomic Deprivation in Virginia in 2020-2021**

**Low-Value** (Rate Ratios; use compared with pre-pandemic rates)

| **Service** | **Quartile** | **2020-2021** |
| --- | --- | --- |
| **Preventive Screenings** | Top ADI quartile (greatest socioeconomic deprivation) | 0.86 |
|  | Lowest ADI quartile (least socioeconomic deprivation) | 1.11 |
| **Diagnostic Tests** | Top ADI quartile (greatest socioeconomic deprivation) | 0.86 |
|  | Lowest ADI quartile (least socioeconomic deprivation) | 1.04 |
| **Pre-Operative Services** | Top ADI quartile (greatest socioeconomic deprivation) | 0.88 |
|  | Lowest ADI quartile (least socioeconomic deprivation) | 0.98 |

**Clinically-Indicated** (Rate Ratios; use compared with pre-pandemic rates)

| **Service** | **Quartile** | **2020-2021** |
| --- | --- | --- |
| **Preventive Screenings** | Top ADI quartile (greatest socioeconomic deprivation) | 0.85 |
|  | Lowest ADI quartile (least socioeconomic deprivation) | 0.91 |
| **Diagnostic Tests** | Top ADI quartile (greatest socioeconomic deprivation) | 0.92 |
|  | Lowest ADI quartile (least socioeconomic deprivation) | 0.93 |
| **Pre-Operative Services** | Top ADI quartile (greatest socioeconomic deprivation) | 0.78 |
|  | Lowest ADI quartile (least socioeconomic deprivation) | 0.81 |

**APPENDIX-J. LOW-VALUE AND CLINICALLY-INDICATED UTILIZATION OF 11 AMBULATORY SERVICES VS. PRE-PANDEMIC RATES IN VIRGINIA DURING 2020 AND 2021 VS. PRE-PANDEMIC RATES, STRATIFIED BY RURALITY**

**REFERENCES**

1. Kind Amy J.H., Buckingham William R. Making Neighborhood-Disadvantage Metrics Accessible — The Neighborhood Atlas. *New England Journal of Medicine*. 2018;378(26):2456-2458. doi:10.1056/NEJMp1802313

2. Mafi JN, Reid RO, Baseman LH, et al. Trends in Low-Value Health Service Use and Spending in the US Medicare Fee-for-Service Program, 2014-2018. *JAMA Network Open*. 2021;4(2):e2037328. doi:10.1001/jamanetworkopen.2020.37328

3. Ganguli I, Morden NE, Yang CWW, Crawford M, Colla CH. Low-Value Care at the Actionable Level of Individual Health Systems. *JAMA Intern Med*. 2021;181(11):1490-1500. doi:10.1001/jamainternmed.2021.5531
